# Extreme Low-Light Image Enhancement for Surveillance Cameras Using Attention U-Net

**DOI:** 10.3390/s20020495

**Published:** 2020-01-15

**Authors:** Sophy Ai, Jangwoo Kwon

**Affiliations:** Department of Computer Science and Engineering, Inha University, Incheon 22212, Korea; sophydfdx@gmail.com

**Keywords:** surveillance camera security, smart city, low-light image enhancement, attention U-net, deep learning

## Abstract

Low-light image enhancement is one of the most challenging tasks in computer vision, and it is actively researched and used to solve various problems. Most of the time, image processing achieves significant performance under normal lighting conditions. However, under low-light conditions, an image turns out to be noisy and dark, which makes subsequent computer vision tasks difficult. To make buried details more visible, and reduce blur and noise in a low-light captured image, a low-light image enhancement task is necessary. A lot of research has been applied to many different techniques. However, most of these approaches require much effort or expensive equipment to perform low-light image enhancement. For example, the image has to be captured in a raw camera file in order to be processed, and the addressing method does not perform well under extreme low-light conditions. In this paper, we propose a new convolutional network, Attention U-net (the integration of an attention gate and a U-net network), which is able to work on common file types (.PNG, .JPEG, .JPG, etc.) with primary support from deep learning to solve the problem of surveillance camera security in smart city inducements without requiring the raw image file from the camera, and it can perform under the most extreme low-light conditions.

## 1. Introduction

Due to a large number of growing smart city projects, surveillance camera security issues in the smart city, especially in night mode or under dark conditions, has become one of the most necessary project implementations. For the past several years, the deep convolutional neural network has achieved great success in many image processing tasks, such as image classification [1,2,3,4,5], image segmentation [6,7,8,9,10], object tracking [11,12,13,14,15], object detection [6,16,17,18,19,20,21], and so on. There is no doubt that the input has to be a high-quality image, and performing the above tasks needs to achieve high performance and good results. Unfortunately, some real images are often degraded under some conditions. For example, an image captured in a low-light environment (owing to the insufficient information received from the scene object) leads to low visibility in such color image scenes, to contrast loss, and to blurred image details. This is a major concern for subsequent computer vision tasks, as well as being an utmost constraint on smart city security development to a great extent, which primarily requires high-quality input images. To tackle these difficulties, a lot of scientific research has examined low-light image enhancement tasks in recent years [22,23,24]. In general, these methods can be grouped into two categories: histogram-based methods and Retinex-based methods. These approaches achieve significant results under certain conditions, but still have some limitations, and underperform in extreme low-light conditions. In this work, we propose a novel low-light image enhancement pipeline based on a deep convolutional neural network. To the best of our knowledge, this work is the first proposing integration between an attention gate and a U-net that is able to perform tasks with normal low-light image files (.PNG, .JPEG, .JPG, etc.) from an extreme low-light dataset (ground truth that corresponds to insufficient image light) to overcome problems with an invisible image under dark conditions. First, we discuss why the Retinex-based method can be replaced with the popular convolutional neural network. The main disadvantage of a Retinex-based method is that, besides learning from data, the parameters of the kernels tend to depend on artificial settings, which leads to reduced accuracy and flexibility in some cases. With motivation by this fact, we selected a deep convolutional neural network instead, because this network can perform and learn directly from end-to-end processing. Second, the histogram-based method is a contrast-enhancement technique designed to have a demonstrated effectiveness. However, slow speeds and overenhancement of noise creates homogeneous regions, which is a problem. The attention model is one of the most influential ideas in the deep learning community, and can be widely utilized in natural image analysis, knowledge graphs, natural language processing (NLP) for image captioning, machine translation, and classification tasks. Self-attention can be used for robust image classification performance by performing class-specific pooling. Overall, our main contributions with this work can be addressed from two aspects: First of all, we extended a self-attention gating module with the standard deep convolutional neural network (U-net), which is able to overcome the needs of raw camera file images, and perform tasks on a common image file, which is easily the future for video processing problems under low-light conditions. Second, the proposed network is an end-to-end network that does not require training the network with different models. Moreover, this research can enable and assist a smart city development to improve the efficiency and sustainability of urban spaces. Specifically, this would allow surveillance camera security (the closed-circuit television (CCTV) camera) be performed under extreme low-light conditions with a better enhancement of image quality and image information results, even if the CCTV camera is installed in a dark area. This also reduces costs, resources, and power consumption.

## 2. Related Work

Over the past years, many researchers have extensively studied the low-light image, and we can provide a short review of the two approaches.

### 2.1. Traditional Approaches

Low-light image enhancement has been actively researched for a long time as an image processing task. There are various traditional techniques, such as histogram equalization (HE) [25], dynamic histogram equalization (DHE) [26], contrast enhancement of low-light images using histogram equalization and illumination adjustment [27], the Retinex-based theory [28], and the multiscale Retinex model (MSR-net) [23]. HE presents as a most popular algorithm for image enhancement because it increases the intensity of the image for better quality. AHE performs the work of transforming the pixel intensity into a proportional display range while also increasing the local intensity histogram, so it will decrease the edges of shadowing problems. These techniques often produce unnatural and unrealistic results, because some priors or assumptions are not good enough to hold for different illumination conditions. Retinex was the early attempt at the Retinex theory–based method, because the image can be recognized from two factors: reflectance and illumination. Multiscale Retinex with color restoration performs reflectance as the final enhancement result, and is used to increase the estimation accuracy of the illumination component by taking advantage of a diversity of complicated filtering methods. However, the performance results usually produced are unnatural, and the image tends to be over-enhanced.

### 2.2. Deep Learning-Based Approaches

Besides the above approaches, there are various studies that extended their work and proposed deep learning-based algorithms to solve these issues. The powerful capability of a deep neural network [3] has led to robust improvements over object recognition. The LLNet proposed in [29] is a deep learning-based method to capture images in low-light environments, which enhances and denoises image captures simultaneously. With the existing deep neural network, LLNet applied the relations between a lightless image and the ground truth enhanced and denoised image. As a result, this deep learning-based method demonstrated suitable performance over low-light image enhancement. Super-resolution [30], MSR-net [24], GLADNet [31], Low-Light Image Enhancement via Illumination Map Estimation (LIME) [32] LIME provides great performance in lightless image problems in which the author develops a structure-aware smoothing model to estimate the illumination map. Google researchers [33] also presented their Night Sight work on the Google Pixel Camera app, which lets us take sharp, clean photographs in very low light in which you can barely see with your own eyes. However, this work is constrained because of its limitation that requires the user to hold the phone for a while to get the raw image bursts, so this process can cause the shaking image problem that so often affects the performance result. From the outstanding results of the U-net network [34] that builds upon a fully convolutional network (FCN) [35], the authors applied the FCN in their network to perform better segmentation in medical imaging. Without a dense layer, the network can be used with images of different sizes. Then, this research work became the winner of the International Symposium on Biomedical Imaging (ISBI) segmentation challenge of 2015. The great success of U-net motivated us to extend their work and apply it to low-light image enhancement problems.

## 3. Proposed Method

**Figure 1:**
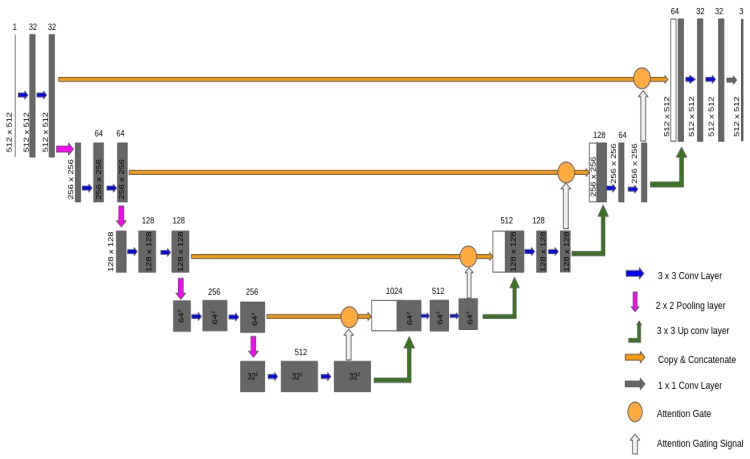
General overview of the proposed Attention U-net.

In the previous section, we discussed the fact that previous work has been conducted to overcome invisible image problems. In this section, we introduce the proposed self-attention-based process, which is used to extend the standard U-net model to generate better performance with low-light image enhancement, and it is called Attention U-net in Figure 1.

### 3.1. Network Architecture

The proposed network architecture in our work was inspired by the standard U-net network [34]. As we mentioned earlier, an attention gate has been used to integrate U-net for robust accuracy improvement with low-light image enhancement problems. We propose a soft-attention technique in a feedforward convolutional neural network (CNN) layer. As seen in Figure 1, this soft attention gate can be worked instead of hard-attention techniques in various computer vision tasks.

#### 3.1.1. Fully Convolutional Network

CNNs outperform traditional approaches in many image processing tasks. We propose using an end-to-end network that is able to perform direct image processing without training under different models. We trained our network model with an FCN [35] to perform our entire image processing network. After a preliminary experiment, we noticed that the fully convolutional network and U-net [34] form the core of our network model. Other proposals explored the residual connection [29], but in our work, we did not find these beneficial results in our setting. Convolutional nets are built on the basic components (convolution, pooling, and activation functions) to perform on the local input regions and relative spatial coordinates. The rectified linear unit (ReLU) has rapidly become the default activation function when developing most types of neural network and use for stochastic gradient descent with backpropagation of errors to train deep neural networks. The feature of the activation function which *f* is a function of input *x* is formed as seen below.

(1) f(x)=max(0,x)

We define loss as a cross-entropy. We can do this because, after upsampling, we got the predictions of the same size as the input so we can compare the acquired segmentation to the respective ground-truth segmentation:

(2) $$C=-\sum_{n=1}^{N}\sum_{k=1}^{K}m_{nk}ln\left(y_{nk}\right)$$

where *N* is a number of pixels, *K* is number of classes, $m_{nk}$ is a variable representing ground-truth with 1 of *K* coding scheme, and $y_{nk}$ represents the predictions (softmax output). While learning parameters, by minimizing them, a training objective like cross-entropy loss measures the performance of a classification model. In this work, we make our attention model on top of a general U-net architecture, whereas the U-net is used to perform image segmentation tasks, because U-net achieves good performance and efficient GPU consumption. Another advantage of using the U-net is to combine the location information from the input image that we applied in downsampling path with the contextual information in the upsampling path to finally obtain a general information combining localization and context, which is important to generate a good segmentation map. As shown in Figure 1, the skip connections are applied with a concatenation operator between the downsampling path and the upsampling path instead of sum. These skip connections intend to allow global information to obtain local information while upsampling. Due to its symmetry, in the upsampling path, the network has a large number of feature maps which allows to transfer information.

#### 3.1.2. Attention Gates in a U-Net Network

**Figure 2:**
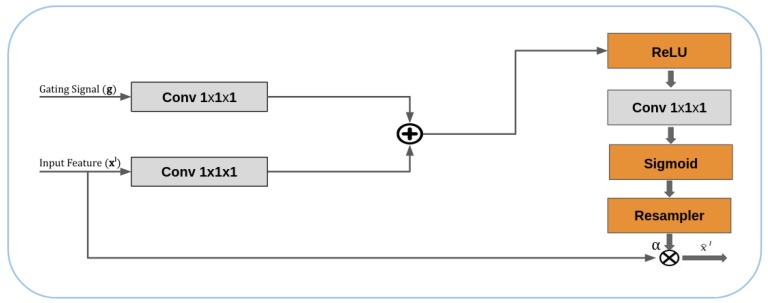
Schematic of attention gates mechanism.

The proposed attention gate (AG) is incorporated into the standard U-net network to enhance model accuracy and sensitivity to foreground pixels while massive computation is not required overhead; also, it does not require training of multiple models and a large number of extra model parameters. This attention gate can steadily decrease features responses in irrelevant background regions. In this research, additive attention gates are implemented through the skip connections before concatenation operation to merge only relevant activations. During the process of backward pass, the gradients originating from background regions are reduced. This enables model parameters in prior layers to be updated depend on spatial regions which are relevant to a given task at each multiscale level. Even additive AGs are more computationally intensive than multiplicative attention, but previous research [36] has illustrated that this additive AG is significantly achieved higher predictive accuracy compared to multiplicative attention. We utilize an adaptation of a previous work [37], which is described additive vector concatenation-based attention method, and this previous work also takes an self-attention approach from [38] and propose grid-based gating, which is more specific to local region to generate a richer representation of attention coefficients, that is, the purpose of the self-attention technique. Figure 2 shows an attention gate has two input feature maps and one element-wise multiplication output of input features and attention coefficient (α result of processing AG) for sequential processing of concatenation operation. The input feature ($x^{l}$) is the output of multiscale encoding convolution block and gating signal (*g*) is spatial regions, which is collected from a coarser scale by analyzing between the activations and contextual information. First, the input feature maps ($x^{l}$) and (*g*) have an individual a 1×1×1 convolutional layer then added together before applying to ReLu activation. Then, 1×1×1 convolutional layer is performed again, but with Sigmoid as activation function layer this time, the sigmoid value has the ranges of [0,1]. After sigmoid, it goes through the resampler layer, which is done by the trilinear interpolation, to create the feature map sizes the same as the one to be element-multiplied. Finally, the concatenation operation can perform with the upsampled feature map at the lower level.

#### 3.1.3. Training

**Figure 3:**
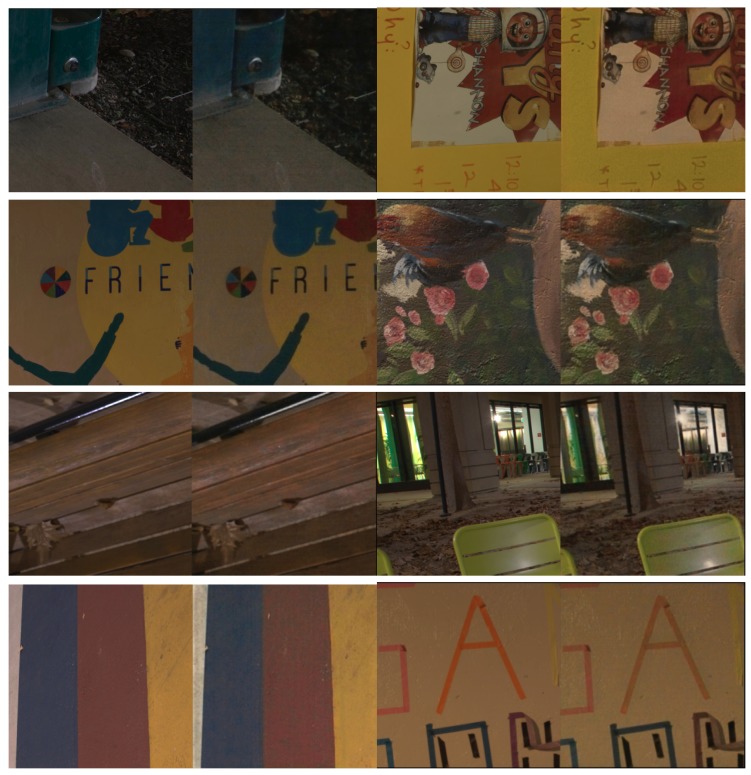
Training result with our converted dataset. On the left side is the ground truth image, and the right side is the result of training the image from a completely dark image.

With integration support from the attention gate with U-net, we successfully trained our network from scratch by using the l1 loss and the Adam optimizer [39] with the See in the Dark (SID) dataset, and we can see some training results in Figure 3. While training, we use the short-exposure image (dark image) as input, which corresponds to a long-exposure image (ground truth image). With every interaction, we randomly crop a 512×512 patch for training, and then, we also apply flipping randomly, and rotation for data augmentation. For the learning rate, we first initialize it to $10^{-4}$ and reduce it to $10^{-5}$ after our learning epochs reach 2000. We trained our network for the whole process over 4000 epochs. The source code [40] is also publicly available on the GitHub.

### 3.2. Dataset

The SID dataset [41] is collection of real-work, extreme low-light images that is included with the corresponding noise-free ground truth images. In the SID dataset, there are 5094 raw short-exposure images in which each image has a corresponding long-exposure reference image. Moreover, the dataset also contains both indoor and outdoor low-light images. Actually, we converted both of the two versions (short-exposed images and long-exposed images from raw format .TIFF files to allow our network to output a .PNG image) without changing the original image size. SID has two dataset versions (Raw Sony and Fuji datasets). Our network model used the Raw Sony data to train the entire network. After converting an SID dataset, the testing process obtains a good quality result from the original images, and we manually made some progress by adjusting a few parameters in the rawpy Python package. We set the amplification ratio to 1000, because we found that our network generates significant results with this number. In Figure 3, we see the training results from the original dataset that was converted to .TIFF files.

## 4. Experiment and Result

**Figure 4:**
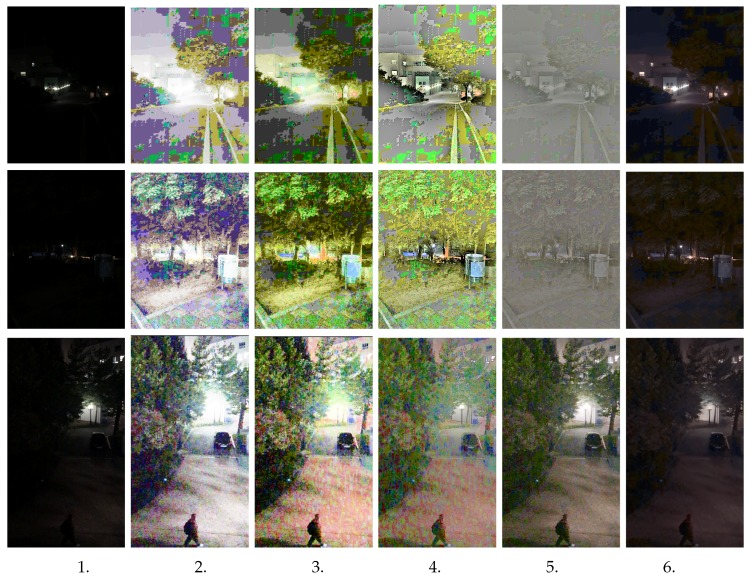
The testing results with an extreme low-light outdoor image from first (left) to last (right): 1. input image, 2. HE [25], 3. DHE [26], 4. Retinex [28], 5. GLADNet [31], and 6. Our proposed method.

**Figure 5:**
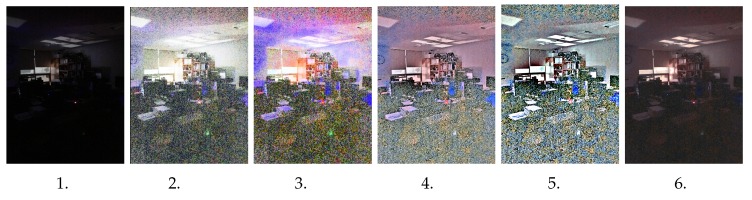
Extreme low-light indoor image results, from first (left) to last (right): 1. input image, 2. HE [25], 3. DHE [26], 4. Retinex [28], 5. GLADNet [31], and 6. our proposed method.

In this section, we evaluate our proposed method for real extreme low-light images. All experiments were performed under Ubuntu 18.04.3 LTS, with 16 GB RAM and an Intel Core i7-5820K CPU @ 3.30GHz with the support of a GeForce GTX 1080 Ti GPU. Our test images were all from real-condition images captured in extreme low-light situations. We compared the results from our proposed method with the most popular traditional methods: histogram equalization and illumination adjustment [25], dynamic histogram equalization [26], Retinex-based theory [28], and deep learning-based GLADNet [31]. We also show the advantages to extending our work as preprocessing to object detection and semantic segmentation in real extreme low-light conditions. As shown in Figure 4 (the outdoor image) and Figure 5 (the indoor image), we illustrate extreme low-light images tested for our network model. It shows our network can perform better, even in an extreme low-light condition, and even the brightness seems to be less than the compared method. However, our network can obtain the natural image color as well as provide noise removal, so please zoom in on the image to see more detail. The traditional methods tend to increase the brightness but fail to restore the natural color of the image, and the noise in the image tends to increase, as well. Although GLADNet on the third test image in Figure 4 can perform a better result, the real extreme low-light conditions (the first and second test images) lost many of the important textures with brightness and natural image color. You can also see the extreme low-light indoor image in Figure 5, where our model strongly obtained the natural color and promised a significant result from noise removal, as well.

## 5. Qualitative Evaluation

For a fair qualitative comparison, we used the three main attributes that are the most favored in image-quality metrics: image quality assessment [42] for peak signal-to-noise ratio (PSNR), the structural similarity metric (SSIM), and multiscale structural similarity for image quality assessment (MS-SSIM) [43] as shown in Table 1. In this comparison, we used the output result from our training model with the real extreme low-light images seen in Figure 4. GLADNet impressively achieved a great performance result, while also performing well based on deep learning. In this paper, we praise their work as state of the art in this low-light image enhancement problem for comparison.

## 6. Discussion

We showed that the proposed Attention U-net can enhance extreme low-light images while also working on noise removal simultaneously, resulting in a significant performance improvement. However, there is still much work to be done and developed. First, even if our network achieved a better enhancement performance compared to the other mentioned methods, our results could not provide a suitable brightness for the output image and, while testing, we have to adjust the amplification ratio manually. It would be useful if we could obtain a good amplification ratio from the input automatically with Auto ISO. Another limitation of our proposed network is that it takes basically 0.56 s to process one 512 × 512 frame; for real-time processing, our proposed network is not fast enough. We expect future research will yield further results and improvements, with great image quality.

## 7. Conclusions

In this paper, we proposed a novel Attention U-net network, which is the integration of a self-attention gate and the standard U-net, and that can be applied to extreme low-light image enhancement, especially for smart city security issues at night time which is possibly saved costs, resources, and power consumption as we reduce the number of street lights, light poles, etc. to surround the camera area in order to make the image environment visible. This work helps enhance an invisible image into a visible image despite low-light conditions by using a common computer vision approach (a deep learning method) without a raw digital camera file (raw file). Moreover, with the proposed architecture, our network is an end-to-end network that is able to perform low-light image enhancement tasks with only the one model. With the results of our work, this proposed network can be extended to a preprocessing model for further research into image processing tasks.

## Figures and Tables

**Table 1:** The qualitative comparison of synthetic extreme low-light images.

Method	PSNR	SSIM	MS-SSIM
HE	6.66	0.28	0.29
DHE	6.77	0.27	0.27
Retinex	8.26	0.12	0.46
GLADNet	10.96	0.18	0.55
Ours	21.20	0.51	0.88
